# Contextual Factors and Mastery Motivation in Young Children with and without Cerebral Palsy: A Systematic Review

**DOI:** 10.3389/fped.2017.00224

**Published:** 2017-10-25

**Authors:** Hsiang-Han Huang, Tzu-Han Sun, Chia-I Lin, Yi-Ru Chen

**Affiliations:** ^1^Department of Occupational Therapy, Graduate Institute of Behavioral Sciences, Chang Gung University, Taoyuan, Taiwan; ^2^Joint Appointment with Department of Physical Medicine and Rehabilitation, Chang Gung Memorial Hospital, Linkou, Taiwan; ^3^Department of Occupational Therapy, Chang Gung University, Taoyuan, Taiwan

**Keywords:** mastery motivation, contextual factors, preschool, cerebral palsy, child development

## Abstract

**Background:**

Mastery motivation is the driving force behind children’s desire to explore the surrounding world and their comprehensive development. However, disease factors may lower a child’s motivation and hamper development. The aim of this review is to examine mastery motivation in preschool children with cerebral palsy (CP) and the impact of contextual factors on mastery motivation.

**Methods:**

Six electronic databases were searched (PubMed, ScienceDirect, Scopus, PsycINFO, Medline, and Airiti Library) using the keywords “Activity,” “Cerebral Palsy,” “Preschool,” “Motivation,” “Mastery motivation,” “Gross motor,” and “Toddler.” We reviewed six observational studies and one interventional study for the following features: (1) participants’ characteristics; (2) assessment, observation, and intervention methods; (3) findings.

**Results:**

Of the seven studies, three were individual cohort studies and four were individual case–control studies. There were two types of motivation-related measures, standardized measurements and observations of structured tasks or free play. Three studies showed no significant difference in mastery motivation between children with and those without CP when given mental-age-appropriate tasks of moderate difficulty. However, environmental factors including social experience, family interaction, and caregivers’ perceptions may affect motivation in preschool children with CP.

**Conclusion:**

Current studies on mastery motivation in preschool children with CP are very limited, and the lack of a universal, theory-based definition of mastery motivation and common assessment frameworks makes it difficult to draw clear conclusions on mastery motivation in children with CP. Future studies should investigate mastery motivation with rigorous study designs to identify ideal activities and environments for preschool children with CP.

## Introduction

Motivation is classified as a mental function which belongs to the body function level in the International Classification of Functioning, Disability, and Health (ICF) (1). As an intrinsic function, mastery motivation enables an individual to autonomously and consistently perform and enjoy activities with moderate difficulty (2, 3). The ICF model emphasizes the dynamic reciprocal relations among functional levels (body function/structure, activity, and participation) and contextual factors (personal and environmental) (1, 4). Mastery motivation, as a function of the body, can not only influence children’s behaviors and performances in both family and educational spheres, but can also increase or decrease as mediated by environmental and personal factors such as family support and age (5, 6). It plays an important role in learning new skills, adapting to new environments, and developing self-efficacy during child development (7). Specifically, studies have found that young children’s developmental abilities are positively correlated with mastery motivation, including toddlers and preschoolers with typical development (TD) (3, 8). Other contextual factors, e.g., the interaction of young children with their primary caregivers, access to objects and materials, age, and gender, may all have effects on children’s mastery motivation (2, 9).

The two primary subtypes of mastery motivation are object (i.e., instrumental) mastery motivation and social (i.e., expressive) mastery motivation (2, 10). The former is represented by persistence and the duration of goal-directed behaviors; the latter refers to enjoyment during or after goal-directed behaviors. Previous studies show that both subtypes of mastery motivation can be investigated with standardized questionnaires, challenging structured tasks, and free play observation (5, 8, 9). They have found mastery motivation emerging in late infancy as a precursor to self-determination, setting a course of increased independence and an enhanced perception of the ability to control one’s environment (2). Starting from the toddler age, the development of mastery motivation is predictable in young children with TD (2, 8). Higher levels of mastery motivation are observed in early life, better developmental outcomes are observed in preschool children with TD. However, disease factors can lower a child’s motivation and subsequently hamper overall development. Cerebral palsy (CP) is one such disease causing motor, cognitive, and sensory disabilities. Global population-based studies estimate that prevalence of CP ranges from 1.5 to more than 4 per 1,000 live births or 1,000 children of a defined age range (11–14). According to the ICF framework, compared with preschool children with TD, preschoolers with CP have poorer health and variable body structures and functions.

Increasing children’s motivation to improve their performance and seek pleasure in activities and participation can be considered a form of intervention, specifically for young children with CP. Current strategies of early intervention mostly utilize top-down approaches and ecological theories to promote the individual’s body functions and health status, e.g., context or function therapy and the application of virtual reality technologies (15–18). These therapies use daily activities as the key intervention, emphasizing motivation of young children with CP to participate in the treatment and then tasking them with functional performances (19). Although targeted standardized tests and assessments of motivation have not been applied in these studies, from a theoretical perspective, the effects of therapy and health status itself would be enhanced if motivation-promoting factors (including autonomy, continuity, and pleasure) were promoted, and environmental and personal factors were considered in children’s activities (3).

The aim of this study is to examine mastery motivation in preschool children with CP and the impact of contextual factors on mastery motivation. A systematic review was conducted to investigate motivation and relevant interventions for preschool children with CP to answer the following questions: Are there differences in motivation between preschool children with and those without CP? Are there contextual factors that affect mastery motivation in children with CP? The results of this review may provide guidelines for clinicians in developing interventions to improve therapeutic outcomes in preschool children with CP.

## Methods

We searched six electronic databases: PubMed (1966 through August 2017), ScienceDirect (1966 through August 2017), Scopus (1966 through August 2017), PsycINFO (1946 through August 2017), Medline (1966 through August 2017), and Airiti Library (1967 through August 2017), using the keywords “Activity,” “Cerebral Palsy,” “Preschool,” “Motivation,” “Mastery motivation,” “Gross motor,” and “Toddler.” References from relevant publications were also included as appropriate. The following inclusion criteria were applied: (1) participants included preschool children with CP (aged under 5); (2) the study included an assessment tool for mastery motivation or detailed discussion on observed changes in motivation; (3) the study was published in a peer-reviewed journal either in English or in Chinese.

**Figure 1:**
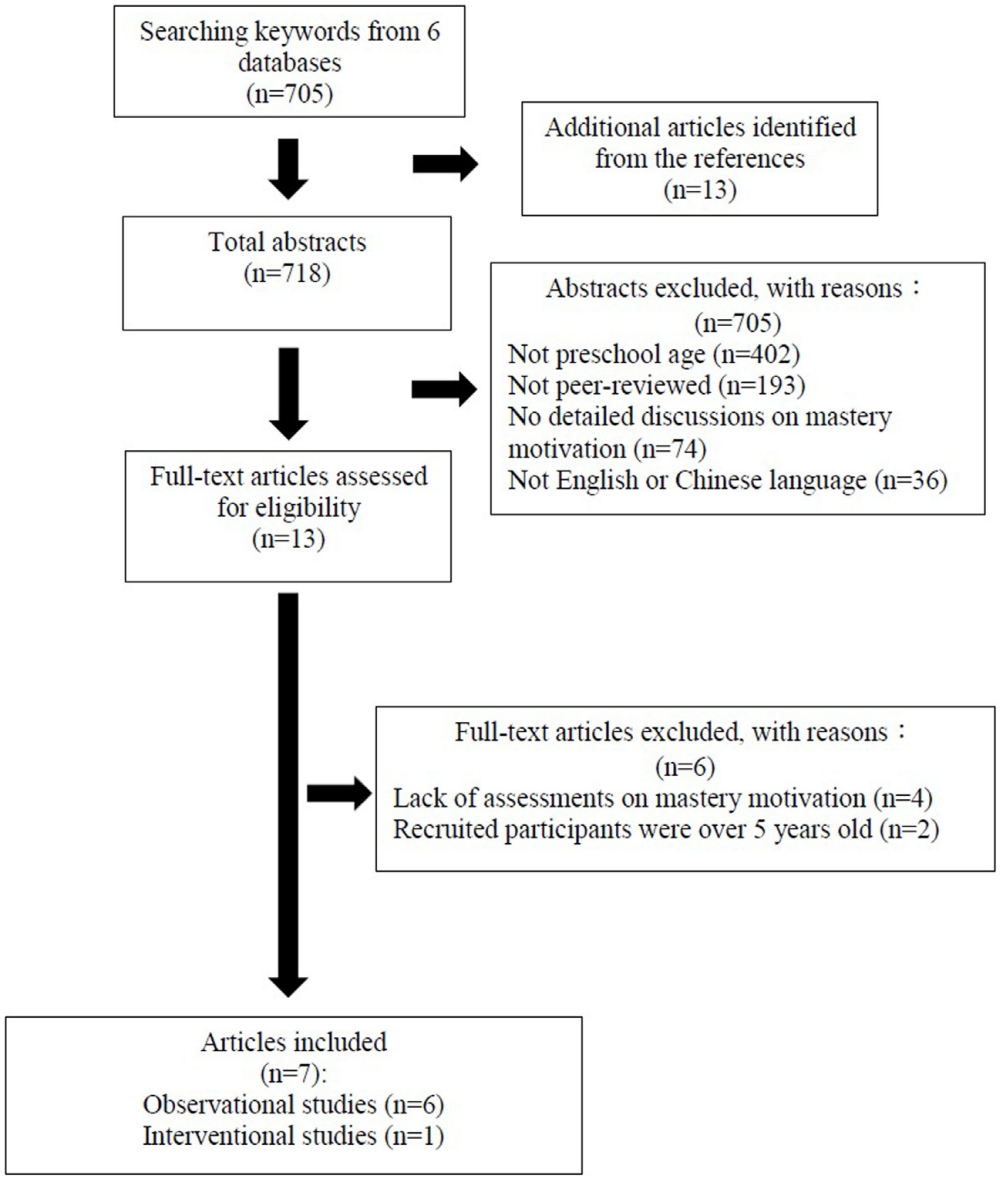
Flow chart of the search results.

Figure 1 shows that 705 articles published till August 2017 were selected. Thirteen more articles were included from references associated with the research topic in this paper, resulting in a total of 718 articles. After title and abstract screening, 595 articles were excluded because the participants were not preschool age or the articles were not peer-reviewed ones. In addition, 74 articles did not provide detailed discussions on mastery motivation and 36 articles were not published in English or Chinese language. 13 articles were chosen by the abstract screening, 6 of which were further excluded after screening of the entire manuscript due to the lack of direct measurements of mastery motivation or because recruited participants were over 5 years of age. Thus, seven articles were included in the analysis. Six were observational studies to examine mastery motivation, and one focused on intervention to promote mastery motivation in children with CP.

## Results

There was limited evidence regarding mastery motivation in preschool children with CP. Seven original studies met the criteria. Using the Oxford Centre for Evidence-Based Medicine’s (OCEBM) levels of evidence classification system (20), the studies offered 2b and 3b levels of evidence (Table 1). Of the seven original studies, three were individual cohort studies (level 2b) and four were individual case-control studies (level 3b). The following features were documented for each study: (1) characteristics of the participants; (2) the assessments and methods of observation or intervention; and (3) findings (Table 2). In the following sections, we have described how these specific features were presented in each study and organized relevant information to elucidate on motivation interventions in preschool children with CP.

**Table 1:** Level of evidence of studies.

Grade of recommendation	Level of evidence	Descriptions	Number of studies	Citations
A	1a	Systematic reviews (with homogeneity) of randomized controlled trials	None	
	1b	Individual randomized controlled trials (with narrow confidence interval)	None	
	1c	All or none randomized controlled trials	None	

B	2a	Systematic reviews (with homogeneity) of cohort studies	None	
	2b	Individual cohort study or low quality randomized controlled trials (e.g., <80% follow-up)	3	Jennings et al. (1988) (23), Waldman-Levi et al. (2015) (25), and Medeiros et al. (2016) (26)
	2c	“Outcomes” Research; ecological studies	None	
	3a	Systematic review (with homogeneity) of case-control studies	None	
	3b	Individual case–control study	4	Jennings et al. (1985) (24), Hauser-Cram (1996) (21), Wang et al. (2013) (7), and Wang et al. (2014) (22)

C	4	Case-series (and poor quality cohort and case-control studies)	None	

D	5	Expert opinion without explicit critical appraisal, or based on physiology, bench research or “first principles”	None	

*Levels of evidence are based on the levels of evidence and grades of recommendations from Oxford Centre for Evidence-Based Medicine (20)*.

*RCT, randomized controlled trial*.

**Table 2:** Summary of studies.

Level of evidence	Citations	Study design	Purpose	Participants	Ages (years)	Observation/intervention	Findings
2b	Jennings et al. (1988) (23)	Observational/cohort with concurrent group	(a) To examine the mastery motivation in children with and without physical impairments at both 3.5 and 4.5 years of age (b) To assess children’s IQ and socioeconomic background to determine equivalence of the two groups of children (c) To examine the relationship between the severity level of impairments and mastery motivation	Total: 61 children with (*n* = 22) and without physical impairments (*n* = 39)(CP: *n* = 12)	Mean age: the first assessment-children with (47 months) and without physical impairments (46 months) The second assessment-children with (59 months) and without physical impairments (58 months)	Structured tasks to assess mastery motivation were administered at school, followed about 2 weeks later by McCarthy Scales of Children’s Abilities. Free play was also observed during this period. The severity level of impairments was rated by the examiners.	Children with TD generally showed more mastery motivation during both structured tasks and free play than their peers with physical impairments. However, there was no significant difference on curiosity. Children with TD persisted more on difficult tasks and more frequently chose challenging tasks over easy tasks. Their activities lasted longer during play and were more complex and cognitively mature; they also spent less tie unfocused. The severity level of impairments showed little relation to motivation. Differences in motivation between these children with TD and physical impairments could be attributed to differences in experiences associated with being physically impairments.

2b	Waldman-Levi and Erez (2015) (25)	Intervention/cohort with concurrent control group	To examine the efficiency of an intervention program for children with developmental disabilities, by modifying both their social and physical environments, in order to enhance their mastery motivation	Total: 19 children with developmental disabilities are assigned to 2 classes (class A: *n* = 9; class B: *n* = 10)(CP: *n* = 12; 6 in each class)	2 to 4Mean age: Class A-41.44 months;Class B-33.8 months	The two classes received two phases of intervention, 6-week social and 6-week physical environmental interventions, in an altering sequence.	The social environment modifications were found to be effective in improving mastery motivation. Moreover, it appeared to have greater improvement than the physical environment modifications. Having supportive, encouraging and sensitive caregivers who promoted children’s mastery motivation was a necessary initial step in treatment planning for these children

2b	Medeiros et al. (2016) (26)	Observational/cohort	To compare longitudinal changes in mastery motivation during parent-child free play for 37 children with complex communication needs	Total: 37 children with complex communication needs(CP: *n* = 16)	9–27 months (CP)	Unprompted parent–child play episodes were identified in three assessment sessions over an 18-month period and coded for 9 categories of mastery motivation in social and object play.	Measuring mastery motivation using social categories such as anticipatory affect or social referencing could provide a less biased representation of mastery motivation for children with relatively low receptive language skills. Low object-based mastery motivation scores reported for children with developmental disabilities may be a function of their impaired motor skills rather than low levels of mastery motivation itself. Encouraging partners to challenge children during social play or adapt object play by adding social elements may be an effective strategy for building and maintaining child mastery motivation in play interactions and reducing common risks for passive interaction styles for children with complex communication needs.

3b	Jennings et al. (1985) (24)	Observational/case–control	To compare the difference on mastery motivation in preschool children with and without disabilities	69 children with (*n* = 25) and without disabilities (*N* = 44)(CP: *n* = 15)	36–53 months (mean: 3 years 10 months)	Motivation in both structured (i.e., structured tasks) and unstructured situations (i.e., free play behavior at school) was observed and assessed. Mothers’ perceptions of their children’s motivation were also assessed.	Intellectual functioning (i.e., IQ) was independent of mastery motivation in children with disabilities. During adult-structured activities, children with disabilities tended to be less persistent on difficult tasks. Moreover, their play during unstructured activities was less complex and peer-oriented. Greater dependency on adults might be one factor affecting the development of mastery motivation in children with disabilities. Children with disabilities might benefit from more unstructured times in which they could develop their own resources and ideas.

3b	Hauser-Cram (1996) (21)	Observational/case–control	(a) To examine the differences on mastery motivation in children with motor impairments, developmental delay and TD (b) To investigate the hypothesized relation between maternal didactic interaction and mastery motivation in children with developmental disabilities	Total: 25 children with typical cognitive development; 25 children with motor impairment (CP) and 25 children with developmental delay, matched for mental age	Mean age (months): Motor impaired: 23.4Developmental delay: 26.0TD: 16.8	A home visit was conducted to assess children’s developmental scores and measure their mastery motivation; mother-child interaction was also observed during a teaching task	Mastery motivation did not differ for young children with delayed or atypical development during sensorimotor period if they were compared to children of a similar level of development and were given tasks of similar difficulty Degree of prematurity, history of a seizure disorder, and maternal didactic interaction were predictive of the measures of mastery motivation in children with developmental disabilities. Children whose caregivers gave clear directions and offered both verbal and nonverbal support and praise when teaching them a task appeared to be more motivated to persist with other challenging tasks on their own.

3b	Wang et al. (2013) (7)	Observational/case–control	(a) To investigate differences between toddlers with and without MD, but matched on mental age and sex, on both the instrumental and expressive aspects of mastery motivation using both the parent-completed questionnaire and behavioral task methods	Total: 22 toddlers with MD; 22 age-matched toddlers with TD(CP: *n* = 4)	24–48 monthsMean age: Children with MD: 30.8 monthsChildren with TD: 21.0 months	Persistence and mastery pleasure were measured with behavioral tasks that were moderately challenging for each child and with maternal ratings using DMQ. Two types of structured tasks (a puzzle and a cause-effect toy selected to be moderately challenging for each child) were administered in a laboratory setting and recorded on video.	The results indicated that the 2 measures assessed different aspects of mastery motivation: parental perception of motivation in everyday life and observations of mastery behavior in a structured setting. Toddlers with MD did not show lower persistence and pleasure when given tasks that were moderately challenging, in comparison with the mental age-matched children with TD. Mothers of toddlers with MD tended to view their children as having low motivation for mastering difficult tasks. For parents’ education, therapists can teach the differences between ability and motivation and the importance of mastery motivation for development.

3b	Wang et al. (2014) (22)	Observational/case–control	(a) To examine the differences in maternal behaviors between toddlers with MD and those with TD (b) To investigate the correlation of maternal behaviors and DQ in toddlers with MD and TD (c) To examine the correlation of maternal behaviors and mastery motivation in toddlers with MD and TD	Total: 22 toddlers with MD; 22 age-matched toddlers with TD(CP: *n* = 4)	Children with MD: 24–47 months (mean: 30.3 months)Children with TD: 15–29 months (mean: 20.7 months)	Mothers and children were invited to the laboratory for a 90-min session. The motor-child teaching interaction observation was conducted and videotaped. CDIIT was administered by a trained pediatric physical therapist. The testing result was used for choosing an estimated initially appropriate difficulty level for later mastery tasks. Subsequently, the child was tested using the individualized structured mastery task method while the mother filled out DMQ in the same room.	Mothers of toddlers with MD exhibited lower cognitive growth fostering behaviors than mothers of toddlers with TD. Maternal total scores were positively correlated with the whole DQ in both groups. Maternal behavior was significantly associated with perceived motivation but not with task motivation in Taiwanese toddlers with MD. Taiwanese mothers of children with MD might be more influenced by having a child with special needs. They might perceive lower mastery motivation of their children even though their children did not exhibit lower task motivation when given tasks that were moderately difficult for them. Mothers of children with MD had lower interactive behaviors compared with mothers of mental age-matched peers with TD.

*CDIIT, Comprehensive Developmental Inventory for Infants and Toddlers; CP, cerebral palsy; DMQ, Dimensions of Mastery Questionnaire; DQ, developmental quotients; MD, motor delay; TD, typical development*.

### Characteristics of Participants

The number of participants ranged from 19 to 69 children at different levels of study (Table 2). Altogether, 255 children aged 9–53 months were included. Eighty-eight (34.5%) children had CP; the remaining 167 had TD, spina bifida, developmental delay, brain injury, and other comorbidities. Six studies required that the participants had developed normal or certain cognitive criteria. For instance, some studies stipulated that children’s mental age be above a certain month (7, 21, 22) or IQ be above 70 (23, 24), or that children display no moderate-to-severe cognitive disability (25). Two observational studies included the Comprehensive Developmental Inventory for Infants and Toddlers (CDIIT) to identify children with movement disorders scoring under 85 (7, 22). The rationale for the cognitive or motor criteria may have been based on the need for each child to perform the structured tasks of motivation tests (9).

### Assessments and Methods of Observation or Intervention

In the seven observational or interventional studies, there were two major types of motivation-related measures, including standardized measurements and observations of structured tasks or free play (Table 3). Standardized measurements included the assessment tool and parents’ questionnaire (7, 22, 24, 25), while observation involved applications of structured tasks with defined psychometric properties (7, 21–24, 26). Three studies used both methods, one used a standardized measurement or questionnaire only, and three used observation only (by an independent person) (Table 3). The standardized measurements included Individualized Assessment of Mastery Motivation, Mother’s Observation of Mastery Motivation (questionnaire), and Dimensions of Mastery Questionnaire. Observations referred to the participants’ performances in completing the structured tasks or during the school free play time and focused on persistence and pleasure. These methods can be used jointly for measuring motivation to provide objective and comprehensive data.

**Table 3:** Summary of studies: outcomes, measures, and results (those relating to cerebral palsy and motivation).

Level of evidence	Study	Outcome of interest	Measure	Result	Statistical significance
2b	Jennings et al. (1988) (23)	Mastery motivation:Structured tasks (a) Persistence at difficult tasks (b) Curiosity (c) Preference for challenging tasks Free play at school (a) Unfocused time (b) Mean duration of play activities (c) Complexity of play (d) Cognitive level of play	Observation Observation Observation Observation Observation Observation Observation	− − − + − − −	Yes No Yes Yes Yes Yes Yes

2b	Waldman-Levi and Erez (2015) (25)	Mastery motivation	Individualized Assessment of Mastery Motivation	+ +	Yes (social environmental intervention) No (physical environmental intervention)

2b	Medeiros et al. (2016) (26)	Mastery motivation:Object-oriented factors (a) Degree of involvement (b) Attention to the task (c) Extent and variety of exploration (d) Persistence Social-oriented factors (a) Anticipatory affect (b) Social interchange with adult (c) Social reference to adult (d) Positive affect (e) Negative affect	Coding scheme adapted from Seifer’s (1996) Mastery Motivation Tasks Scoring Manual Observation Observation Observation Observation Observation Observation Observation Observation Observation	No change over time	No No No No No No No No No

3b	Jennings et al. (1985) (24)	Mastery motivation:Structured tasks (a) Persistence at difficult tasks (b) Curiosity Free play at school (a) Attention span (b) The complexity of play (c) The degree of involvement (d) The level of social participation Mothers’ perceptions of children’s mastery motivation: (1) General mastery motivation (2) Preference for easy and familiar tasks (3) Need for adult help or approval (4) Need for adult structure (5) Resistance to adult direction	Observation Observation Observation Observation Observation Observation Mother’s Observation of Mastery Motivation (36-item questionnaire)	− + + − − − − + + + −	Yes No No Yes Yes Yes Yes Yes No No No

3b	Hauser-Cram (1996) (21)	Mastery motivationTwo-problem posing tasks (a) Persistence – cause-effect tasks – puzzle tasks (b) Non-goal-oriented manipulation – cause-effect tasks – puzzle tasks (c) Competence – cause-effect tasks – puzzle tasks (d) Positive affect – cause-effect tasks – puzzle tasks	Problem-posing mastery motivation measures Observation Observation Observation Observation Observation Observation Observation Observation	+ − − + − − − −	No No No No No No No No

3b	Wang et al. (2013) (7)	Mastery motivation:Caregivers’ perceptions: (a) Total persistence (b) Mastery pleasure Individualized structured mastery tasks (a) Task persistence (b) Task pleasure	Dimensions of Mastery Questionnaire (DMQ)Individualized structured mastery tasks Observation Observation	− − + +	Yes Yes No No

3b	Wang et al. (2014) (22)	Mastery motivation:Caregivers’ perceptions: Instrumental aspects (c). Object-oriented persistence (d). Gross motor persistence (e). Social persistence with adults (f). Social persistence with children (g). Total persistence Expressive aspects (a) Mastery pleasure (b) Negative reaction to failure The child’s ability (a) General competence Individualized structured mastery tasks (c) Task persistence – puzzle – cause-effect (e) Continuity of task engagement – puzzle – cause-effect (g) Mastery pleasure – puzzle – cause-effect	DMQ Individualized structured mastery tasks Observation Observation Observation Observation Observation Observation	− − − − − − + − + + + + No difference	Yes Yes Yes Yes Yes No No Yes No No No No No

Some studies reported detailed observations of the following: positive emotions during activities (such as competence, pleasure, interest, and self-efficacy), duration of activities, initiation of participation, willingness to participate in activities, and length of children’s attention maintained during an activity. These indices are included in the definition of mastery motivation (10). In addition, in two studies, assessments of children’s curiosity during activities and interactions with parents during activities were also considered (23, 24). In six observational studies, the children were asked to conduct structured tasks or carry out free play with the therapist or researcher observing their interactions with parents (7, 21, 22, 26) or peers (23, 24). The only motivation-related interventional study aimed to modify the social and physical environments to enhance children’s mastery motivation (25). The interventions were based on the guidelines described in the Early Childhood Environment Rating Scale-Revised (ECERS-R) for improved environments and included furniture and space arrangement, creation of quiet area and interest areas, room arrangement facilitating children’s autonomy, free play and child’s independence, verbal mediation, and verbal reinforcement.

### Research Findings

Factors influencing motivation for preschool children with CP were discussed in five observational studies and compared with those influencing children with TD (Tables 2 and 3). Specifically, two of them reported that preschool children with disabilities tended to show less mastery motivation and persistence on tasks than children with TD while interacting with peers, but intellectual functioning was independent of mastery motivation (23, 24). However, as reported by Hauser-Cram (21), cognitive functions can affect the level of motivation in children with CP. Three studies showed that there was no significant difference in mastery motivation between children with atypical development and TD while interacting with parents (7, 21, 22). In addition, studies indicated that several factors associated with physical impairments, social play, degree of prematurity, history of a seizure disorder, maternal didactic interaction, and caregivers’ perceptions of children’s motivation may influence preschooler’s mastery motivation (7, 21–23, 26). Jennings et al. (23) reported that motivation of preschool children increased with age; however, social experience, which varied with different cognition degrees, significantly contributed to the development of motivation. The findings of the five studies relating to the observations during structured or free play indicated the social experience from the interaction with parents or peers might result in different effects on mastery motivation (7, 21–24, 26); the positive interaction between parents and the child might promote his/her mastery motivation which is similar to the child with TD. Wang et al. (7, 22) also reported that if children with and those without developmental delay were assigned tasks corresponding to their mental age and with moderate difficulty, their motivation for mastery was the same. Task difficulty was a crucial factor in motivation assessment. In addition, caregivers’ perceptions and interaction styles also greatly influenced children’s motivation. One study compared different modes of interaction between mothers and children and concluded that didactic interactions could promote children’s motivation (21). Another pointed out that children with CP were likely to be overly dependent on their caretakers, which was detrimental to the development of motivation (24).

In terms of intervention, Waldman-Levi and Erez (25) reported that a combination of physical and social modifications may be the best way to improve mastery motivation in children with CP, although the social environment modifications appeared to have a greater effect than the physical environment modifications. Moreover, they found that encouraging, supportive, and sensitive caregivers played an important role in promoting children’s mastery motivation in the initial phase of treatment planning.

Overall, most observational studies found that preschool children with CP showed similar persistence and pleasure as children with TD. However, the studies identified several factors that may influence mastery motivation, including measures of motivation (i.e., object or social mastery motivation), task difficulty, parenting styles, caregivers’ perceptions of children’s mastery motivation related to cultural contexts, and children’s experience of physical impairment. Relevant interventions focusing on modifying physical and social environments may be beneficial for improving both object and social mastery motivation in preschool children with CP.

## Discussion

To date, this is, to our knowledge, the first systematic review to examine and compare mastery motivation in preschool children with and those without CP and to provide evidence on motivation-related interventions in this particular pediatric population. The seven relevant studies we found show that there may be no significant difference in mastery motivation between children with and those without CP when administered tasks of moderate difficulty and suitable mental age. However, contextual factors, particularly environmental factors, including social experience, family mode of interaction, and caregivers’ perceptions, can affect the level of motivation in preschool children with CP. Notably, there are few studies on preschool children’s motivation, most of the available evidence is low-level—implying that the field of contextual factors and mastery motivation in young children with CP is under-researched—and large-scale rigorous research designs are scarce. Thus, future studies should focus on examination of these factors to provide more detailed information.

It remains unclear whether mastery motivation varies with cognitive functional level in preschool children with CP. The included studies had conflicting results; one study reported that cognitive function had no effect on motivation level (24), while another reported a contradictory result, while also reporting that differences in motivation of children with and those without developmental delay was negligible before the sensorimotor stage and only became significant afterward; however, this needs further explanation (21). Although both articles considered similar factors in their assessments, contrasting results were obtained, and no consistent conclusion was reached. This may be because early studies did not have a clear definition of motivation, and assessment methods used in the studies were different. Three studies proposed that children with and those without CP have similar motivation when administered tasks of moderate difficulty (7, 21, 22). Therefore, the suitability of a task may be more important to motivation than cognitive functions (2).

Among studies reviewed here, assessment tools, including standardized tools, were often used in conjunction with observation by a third person, e.g., a therapist or a teacher, because of their objectivity and professionality. In studies involving observations by both parents and therapists, mothers were inclined to judge the motivation level by the level of competence in their children; for example, mothers of children with disabilities tended to think that their children had low motivation levels (3, 5). Parents may consider tasks too difficult for their children and underestimate their children’s motivation in questionnaires; a disadvantage potentially limited by parents’ perceptions of children’s capabilities (27, 28). However, parental observation has advantages as well because it offers data on children’s performances in a natural environment and reduces the cost and time of studies (7, 9). In summary, observations from both therapists and parents are recommended to further understand changes in motivation and influencing factors for preschool children. Before parents complete the questionnaires, therapists should educate them on the concept of motivation and the suitability of activities for their children (7, 29).

Toward operationalizing a definition of mastery motivation, studies included observation of persistence, attention span, curiosity, competence, pleasure, and positive and negative pleasure among their measures. Many studies mentioned the impact of motivation on overall development in children with CP, specifically for upper and lower limb motor development (17, 18, 30–32). However, there was a lack of shared definition of motivation and assessment tools to detect changes in motivation before and after an intervention. For instance, some studies emphasized improvements in children’s performances but regarded the increase in movement frequency and function as indicators of motivation (31, 32). The studies employing virtual reality technologies also focused on the role of motivation in shaping motor outcomes (17, 18). These studies used standardized assessments to measure children’s playfulness and the VR system to record children’s drive to play and intensity of play during an intervention. This method may overlook the definition of mastery motivation and the challenging level of the games is mostly set up by the program, not according to the child abilities (33). Future studies will benefit from a standardized approach of measuring motivation, its relationship to environmental factors, and the impact of interventions. We suggest that developing such an approach is a priority for researchers and practitioners.

According to Turner and Johnson’s (3) proposed model of mastery motivation, children with high mastery motivation will engage with toys, people, and events, and mastery motivation can be divided into object and social mastery motivation (2). Furthermore, parenting beliefs and parent–child relationships may influence these two subtypes of mastery motivation (3, 5, 34). Previous studies have found that parent-child relationships were significantly positively linked to mastery motivation in preschool children with CP (3, 21, 22, 25). Waldman-Levi and Erez (25) also suggested that encouraging, supportive parents may promote children’s mastery motivation in physical and social environments during an intervention. Moreover, parents’ self-efficacy was directly linked to parents’ beliefs and parent–child relationships (3), in that parents with high self-efficacy seem to have more positive beliefs and better relationships with their children, thereby potentially supporting their children’s greater mastery motivation. Thus, therapists should carefully structure the environment, work with primary caregivers to enhance their self-efficacy, and teach methods of improving parent-child relationships (3, 4). Of note is that two studies involving interactions with peers showed less mastery motivation and persistence on tasks in children with CP than children with TD (23, 24). Although the evidence is very limited, this may indicate the interaction with other children will result in different social experience on mastery motivation. Future studies may include the interactions with parents or other children as one environmental factor and compare their effects on mastery motivation, which may provide guidelines for future motivational interventions. In addition, adding social elements to object play may be an effective way to induce or maintain children’s mastery motivation and increase active interaction styles in play (26). In summary, according to the ICF framework, different functional levels can interact and further influence health. Thus, in further investigations of mastery motivation in preschool children with CP, we suggest that a variety of assessment tools be applied and targeted assessments of relationships between body function, activity, and participation levels be carried out. Clinicians can also discuss training for caregivers in promoting children’s autonomy, continuity, and pleasure in daily activities and social and environmental influences on children’s motivation.

## Limitations

The evidence included in this study are only those published in English; other appropriate studies in other languages may have been ignored. In addition, although an extended search was performed by combining various keywords, studies that used different terminology for mastery motivation may have been inadvertently excluded due to the inclusion criteria of direct assessment and explicit discussion of motivation in the studies. Furthermore, in some studies, children with diagnoses other than CP were included; therefore, the results only covered a small group of preschool children with CP and cannot be generalized to the overall population.

## Conclusion

For preschool children, mastery motivation is the driving force behind the desire to explore the surrounding world and is necessary for comprehensive development (2, 4, 10). According to the ICF model, motivation is a function of the body and can be shaped by environmental and personal factors. Thus, it is essential to identify the optimal physical and social environments for children’s participation in activities and to effectively improve children’s mastery motivation and therapy outcomes. However, current studies on the mastery motivation of preschool children with CP are very limited, and the lack of a universal, theory-based definition and assessment methodology makes it difficult to answer key questions about this population. It is therefore suggested that mastery motivation be further investigated with more rigorous study designs, with the objective of identifying ideal activities and environments for preschool children with CP.

## Author Contributions

H-HH is responsible for the acquisition, analysis, interpretation of data, drafting, and revising the work. T-HS, C-IL, and Y-RC are responsible for the acquisition, analysis, and drafting the work.

## Conflict of Interest Statement

The authors declare that the research was conducted in the absence of any commercial or financial relationships that could be construed as a potential conflict of interest.
